# Inhibition of γδ-TcR or IL17a Reduces T-Cell and Neutrophil Infiltration after Ischemia/Reperfusion Injury in Mouse Liver

**DOI:** 10.3390/jcm12051751

**Published:** 2023-02-22

**Authors:** Saant Al Mogrampi, Christina Boumpoureka, Hara Afaloniati, Maria Lagou, Katerina Angelopoulou, Doxakis Anestakis, Zoi Gerasimina Tampouratzi, Stavros Iliadis, Nikolaos Antoniadis, Alexandros Giakoustidis, Apostolos Papalois, Vasileios Papadopoulos, Theofilos Poutahidis, Dimitrios Giakoustidis

**Affiliations:** 1 Medical School, Faculty of Health Sciences, Aristotle University of Thessaloniki, 54124 Thessaloniki, Greece; 2 Department of Surgery, General Hospital of Imathia, Naousa Health Unit, 59200 Naousa, Greece; 3 Liver Unit, Institute of Liver Studies, King’s College Hospital, London SE5 9RS, UK; 4 Laboratory of Biochemistry and Toxicology, School of Veterinary Medicine, Faculty of Health Sciences, Aristotle University of Thessaloniki, 54124 Thessaloniki, Greece; 5 Laboratory of Pathology, School of Veterinary Medicine, Faculty of Health Sciences, Aristotle University of Thessaloniki, 54124 Thessaloniki, Greece; 6 Laboratory of Pathology, Forensics Department, Ministry of Justice, 56334 Thessaloniki, Greece; 7 Department of Biological Chemistry, Medical School, Faculty of Health Sciences, Aristotle University of Thessaloniki, 54124 Thessaloniki, Greece; 8 Department of Transplant Surgery, Medical School, Faculty of Health Sciences, Aristotle University of Thessaloniki, G. H. Hippokration, 54124 Thessaloniki, Greece; 9 Department of Surgery, Medical School, Faculty of Health Sciences, Aristotle University of Thessaloniki, G.H. Papageorgiou, 54124 Thessaloniki, Greece; 10 Experimental, Educational and Research Center (ELPEN), Pikermi, Rafina-Pikermi, 19009 Athens, Greece; 11 Special Unit for Biomedical Research and Education, School of Medicine, Aristotle University of Thessalniki, 54124 Thessaloniki, Greece; 12 School of Medicine, European University Cyprus, Engomi, 2404 Nicosia, Cyprus

**Keywords:** γδ-Τ cells, Th17, Treg, liver surgery, hepatic injury, ischemia/reperfusion injury, liver transplantation

## Abstract

Neutrophil and T-cell recruitment contribute to hepatic ischemia/reperfusion injury. The initial inflammatory response is orchestrated by Kupffer cells and liver sinusoid endothelial cells. However, other cell types, including γδ-Τ cells, seem to be key mediators in further inflammatory cell recruitment and proinflammatory cytokine release, including IL17a. In this study, we used an in vivo model of partial hepatic ischemia/reperfusion injury (IRI) to investigate the role of the γδ-Τ-cell receptor (γδTcR) and the role of IL17a in the pathogenesis of liver injury. Forty C57BL6 mice were subjected to 60 min of ischemia followed by 6 h of reperfusion (RN 6339/2/2016). Pretreatment with either anti-γδΤcR antibodies or anti-IL17a antibodies resulted in a reduction in histological and biochemical markers of liver injury as well as neutrophil and T-cell infiltration, inflammatory cytokine production and the downregulation of c-Jun and NF-κΒ. Overall, neutralizing either γδTcR or IL17a seems to have a protective role in liver IRI.

## 1. Introduction

Ischemia/reperfusion injury (IRI) occurs when the blood supply to an organ is temporarily disrupted, followed by the restoration of normal blood flow. Liver transplantation, liver resection with clamping of the portal triad and low flow states, such as hypovolemia, cardiogenic shock and sepsis, can be major causes of IRI [1]. Hepatic inflammation due to IRI is multifactorial and can last up to a week after reperfusion [2]. Soon after reperfusion, an inflammatory cascade orchestrated by Kupffer cells occurs, exacerbating reactive oxygen species (ROS) and cytokine production. Subsequently, neutrophils, macrophages and various subsets of effector CD4+ T cells migrate to the liver parenchyma reinforcing the inflammatory response [3,4]. Although CD4 lymphocytes were considered “innocent bystanders”, data have emerged indicating their importance in IRI and trauma [5]. It appears that CD4+ T cells migrate to the liver within an hour of reperfusion and play a pivotal role in neutrophil recruitment [6,7,8]; however, the precise mechanism remains unclear [9].

IL17a is a proinflammatory cytokine, previously believed to be almost exclusively produced by activated CD4+ lymphocytes, especially Th17 cells [10]. The IL17a blockade has been shown in previous studies to reduce I/R injury by reducing neutrophil recruitment and suppressing cytokine and adhesion molecule production [11]. γδ-T cells are a subcategory of lymphocytes, whose TcR consists of γ and δ chains, and make up one-quarter of liver lymphocytes—a much higher proportion than in peripheral blood [5]. Being on the verge of innate and adaptive immunity, they are not MHC-restricted and recognize molecular signals related to cellular damage while also exhibiting a diversity of their TcR and adaptive clonal expansion in certain cases [12]. They are considered to have both effector and regulatory functions, while their role in liver inflammation has not yet been fully elucidated [13]. 

PPAR-γ is a nuclear transcription factor implicated in promoting the anti-inflammatory phenotype of macrophages and reducing inflammatory response [14] and has been suggested to attenuate liver IRI in animal models. The exact mechanism is unclear; however, the activation of PPAR-γ seems to be a protective cellular response after liver IRI [15], while its downregulation increases hepatocyte injury [14]. NRF2 is a transcription factor that has been implicated in redox homeostasis, inflammation and apoptosis [16]. It has been shown to play a protective role in liver IRI, mainly by inducing the transcription of antioxidant genes in response to increased intracellular ROS [17,18] and by reducing necrosis and apoptosis after liver IRI [19].

In the present study, we used in vivo pretreatment with anti-γδTcR and anti-IL17a antibodies before I/R induction in order to investigate whether a γδTcR and IL17a blockade results in the amelioration of IRI, downregulation of proinflammatory transcription factors, reduction in neutrophils and recruitment of T cells in mouse liver.

## 2. Materials and Methods

### 2.1. Experimental Procedure

Forty male C57BL6, eight-week-old mice, 24–26 g, were used. The mice were cared for in cages, housed in the experimental facilities of the ELPEN laboratory (EL 09 BIO 03), in accordance with best practice standards in animal handling (Guide for the Care and Use of Laboratory Animals) [20], at room temperature (20–22 °C), with a 12 h light–dark cycle. They had free access to food and water. Experiments were approved by the relevant Ethics Committee and were compliant with European Union and National Law (Articles 35-44/PD 56/2013) (RN 6339/2/2016).

The mice were randomly divided into 4 groups of 10; two control and two experimental groups as follows:

1. Sham group. Animals were anaesthetized and subjected to a sham surgical operation that involved a midline incision equivalent to the experimental groups but with no ischemia time;
2. I/R group. Animals were subjected to partial hepatic ischemia for 60 min followed by 6 h of reperfusion;
3. I/R + anti-γδTcR group. Animals were pretreated with an intraperitoneal injection of 500 μg of anti-γδTcR antibody [21] (Purified NA/LE Hamster anti-mouse γδ-Τ-cell Receptor, UC7-13D5, Catalog Number: 553181, BD PharmingenTM, San Jose, CA, USA) 72 h before induction of ischemia;
4. I/R + anti-IL17 group. Animals were pretreated with a 100 μg anti-IL17a antibody [22] (Mouse IL-17/IL -17A Antibody Monoclonal Rat IgG2A Clone # 50104, Catalog Number: MAB421) injection into the inferior vena cava (IVC) before induction of ischemia.

The animals were anaesthetized using an intraperitoneal injection consisting of a mixture of 100 mg/kg ketamine and 6.7 mg/kg xylazine. Repeat doses of ketamine were used as needed. Intraperitoneal heparin 50 IU/kg was administered as prophylaxis against venous thromboembolism. Throughout the operation, the animals were placed on a heated pad at constant temperature and had a continuous O_2_ supply.

A model of in vivo warm ischemia widely established in the literature was used, as described by Kono et al. [11] and Lentsch et al. [23]. We induced partial liver ischemia to the left lateral lobe and median lobe, with clamping of the portal triad distal to the branch supplying the right lobe of the liver with an atraumatic vascular microclip (Micro VASCU-STATT, 1001-499, Scanlan International, St. Paul, MN, USA). The microclip was removed 60 min later and blood flow to the liver was restored. The midline laparotomy was sutured and animals were allowed to recover in their cages. After 6 h of reperfusion, animals were anaesthetized and the midline opened. Blood samples were obtained with cardiac catheterization and the middle and left liver lobes were removed for sampling.

### 2.2. Histopathology, Immunohistochemistry and Morphometry

For histopathologic analysis, formalin-fixed liver lobes were embedded in paraffin, sectioned at 4–5 μm, and stained with haematoxylin and eosin or immunohistochemistry (IHC). The I/R-induced pathology in the liver was scored on a 0–4 scale of ascending extent and severity using previously described criteria [24,25].

Primary antibodies for IHC included rabbit antibodies against myeloperoxidase (MPO), PPAR-γ (RB-373 and K.242.9, ThermoFisher Scientific/Lab Vision, Fremont, CA, USA), Foxp3 (ab215206, Abcam, Cambridge, UK), NF-κB-p65, c-Jun (#8242 and #9165, Cell Signalling, Beverly, MA, USA), IL-32 (SP7196P, Acris Antibodies GmbH, Herford, Germany) and CD3 (103A, Cell Marque, Rocklin, CA, USA). Heat-induced antigen retrieval was performed with a citrate buffer, pH 6, for myeloperoxidase (MPO), NF-κB-p65, c-Jun and IL-32, and with CC1 epitope retrieval solution for CD3 (950-124, Ventana Medical Systems, Inc., Oro Valley, AZ, USA). Antibodies were diluted at 1:100 in antibody diluent OP Quanto (TA-125-ADQ, Epredia, Breda, Netherlands). Rabbit primary antibody binding was detected with goat anti-rabbit polymer HRP (ZUC032, ZytoChem Plus, Berlin, Germany). The colour was developed with Diaminobenzidine substrate-chromogen (HK124/HK520, Biogenex, Fremont, CA, USA) and tissue sections were counterstained with haematoxylin.

For quantitative histomorphometry, IHC-positive immune cells or pixels were counted in images of ×40 or ×10, using representative high-power fields, as previously described [25], and results were recorded as the number of cells or pixels per image. ImageJ image processing and analysis software (NIH, Bethesda, MD, USA) was used for all quantitative histomorphometry assessments.

### 2.3. Quantitative Gene Expression Analysis

Total RNA was extracted from tissue samples using the NucleoSpin Total RNA Isolation kit (740,955.50, Macherey-Nagel, Duren, Germany) according to the manufacturer’s instructions. After the spectrophotometric determination of RNA concentration and quality, samples were stored at –80 °C until use. Reverse transcription was carried out using the FastGene Scriptase II cDNA kit (LS65, Nippon Genetics, Japan). Five hundred ng of total RNA was used as the starting material for cDNA synthesis. Real-time PCR based on the SYBR Green chemistry was used to quantitatively analyze the expression of the genes, as shown in Table 1. The housekeeping gene glyceraldehyde-3-phosphate dehydrogenase (GAPDH) was used as an internal control. Primers were designed using Primer3 Input software (version 0.4.0; Whitehead Institute for Biomedical Research, Cambridgeu, MA, USA) according to nucleotide sequences available in GenBank. Primer sequences, their positions within the corresponding genes, GenBank accession numbers and amplicon sizes are presented in Table 1. PCR amplification was performed in 10 μL reaction mixtures containing 2 μL of cDNA, 1× KAPA SYBR FAST qPCR master mix (KK4602, KAPA BIOSYSTEMS, Woburn, MA, USA), and 100–300 nM of each primer pair (Table 1). The temperature cycling on a PCRmax Eco 48 real-time PCR system (PCR max, Staffordshire, UK) included 35–40 cycles consisting of denaturation at 95 °C for 10 s and annealing/extension at temperatures ranging from 59 to 63 °C for 20 s (Table 1).

Each PCR reaction was initiated with a 3 min denaturation at 95 °C and terminated with sequential readings between 65 and 95 °C (increment 0.5 °C) in order to generate the melting curve and verify amplicon specificity. For the relative quantification of gene expression, we used the comparative Ct method, also known as the 2^−ΔΔCt^ method [26].

### 2.4. Determination of Malondialdehyde (MDA) in Liver Tissues

The determination of MDA, the compound used as an index of lipid peroxidation, was carried out with a selective third-order derivative method [27].

In brief, 0.1 g of the tissue samples were thoroughly homogenised (Polytron homogenizer, PCU, Zurich, Switzerland) with 5 mL of 5% aqueous trichloroacetic acid; 2 mL of 0.8% butylated hydroxytoluene in hexane were added to the homogenate and centrifuged. The top layer was discarded, and a 2.5 mL aliquot from the bottom layer was mixed with 1.5 mL of 0.8% aqueous 2-thiobarbituric acid to be further incubated at 70 °C for 30 min. Following incubation, the mixture was cooled to room temperature and submitted to conventional spectrophotometry (Model UV-160A, Shimadzu Corp., Tokyo, Japan) in the range of 400–650 nm with a scanning speed of 480 nm/minute. Third-order derivative spectra were obtained by electronic differentiation (using a derivative difference setting of 21 nm) of the conventional absorption spectra of samples from both control and drug-treated mice. MDA concentration (nmol/g of wet tissue) was calculated based on the third-order derivative peak height at 532 nm by referring to the slope and intercept data of the computed least-squares fit of the standard calibration curve.

### 2.5. Statistical Analyses

The histopathological scores, morphometric counts, relative gene expression and MDA concentration data were compared between groups using Mann–Whitney U analysis. Statistical significance was set at *p* < 0.05. All analyses were performed with GraphPad Prism version 8.0.1 for windows (GraphPad Software, San Diego, CA, USA). Data were represented as bar graphs depicting the mean and standard error of the parameter assessed for each experimental group.

## 3. Results

### 3.1. Depletion of Either γδ-T Cells or IL17 Ameliorates I/R-Induced Liver Injury

**Figure 1:**
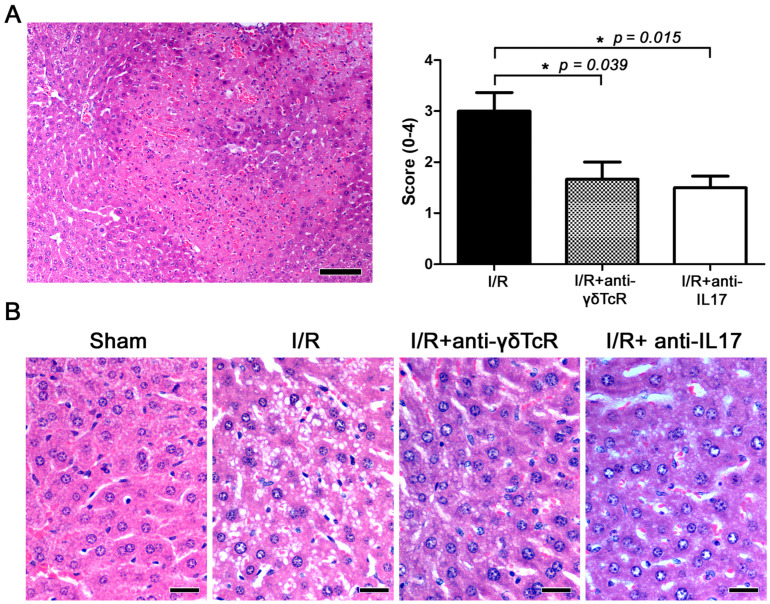
Depletion of γδTcR cells or IL17a ameliorates I/R-induced liver injury in mice. (**A**) Representative area with bridging submassive coagulative necrosis area in mouse liver after I/R. The necrotic area of liver tissue shows pale eosinophilic stain, hepatocyte loss, hyperhaemia and multifocal haemorrhages and typical cell nuclear changes in necrosis. Histopathological scores of I/R injuries given to the different experimental groups are shown in the bar graph on the right. Pretreatments significantly ameliorated liver histopathology. (**B**) Side-by-side comparison of typical hepatocyte histology in the four experimental groups of mice. Compare normal hepatocytes of sham-operated controls with hepatocytes showing cytoplasmic vacuolar degeneration in the I/R-treated mice. Degenerative lesions are milder after the neutralization of γδTcR cells or IL17a. (**A**,**B**) Haematoxylin and Eosin. Scale bars: 100 μm (**A**) and 25 μm (**B**). (**A**) Numbers on the Y-axis of the bar graph correspond to the mean ± SEM of the histopathological score; * *p* < 0.05.

The liver lobes of mice that underwent experimental I/R had the standard array of I/R-induced histopathological lesions, including occasional areas of massive necrosis, multifocal congestion, hepatic cell vacuolar degeneration and necrosis (Figure 1A,B). The same liver lobes of sham-treated mice had normal histology (Figure 1B). The livers of mice that were pretreated with anti-γδ-TcR or anti-IL-17 antibodies before I/R induction showed ameliorated histopathology in comparison with their nonpretreated counterparts. To further support this observation, we scored the severity and extent of histopathological lesions in the mouse livers. The statistical analysis of histopathology scores supported our observation by yielding statistically significant effects of both anti-inflammatory treatments in suppressing IRI (Figure 1A,B).

**Figure 2:**
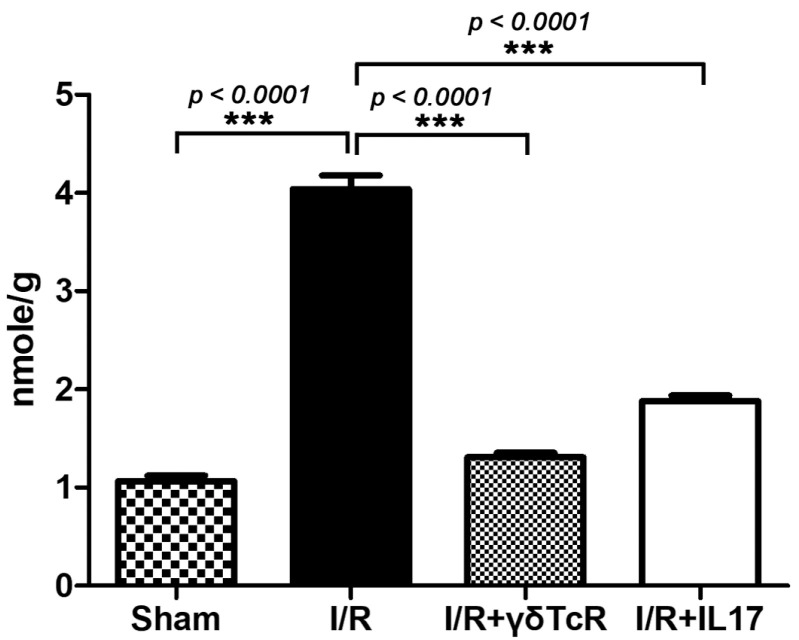
Liver biochemistry for MDA reflects the significant effect of both anti-inflammatory pretreatments in reducing I/R-induced injury. Numbers on the Y-axis of the bar graph correspond to the mean ± SEM of MDA concentration. *** *p* < 0.001.

To confirm this result, we next determined levels of MDA, a well-accepted index of lipid peroxidation and cell injury, in the livers of mice. Liver biochemistry results were in line with pathology since there was a statistically significant MDA elevation in I/R mice compared to the sham-operated controls. Furthermore, the neutralization of either γδTcR or IL17a prior to I/R reduced MDA at statistically significant levels (Figure 2).

### 3.2. Anti-γδ-TcR and Anti-IL17 Treatment Reduce I/R-Induced Inflammatory Cell Infiltration

**Figure 3:**
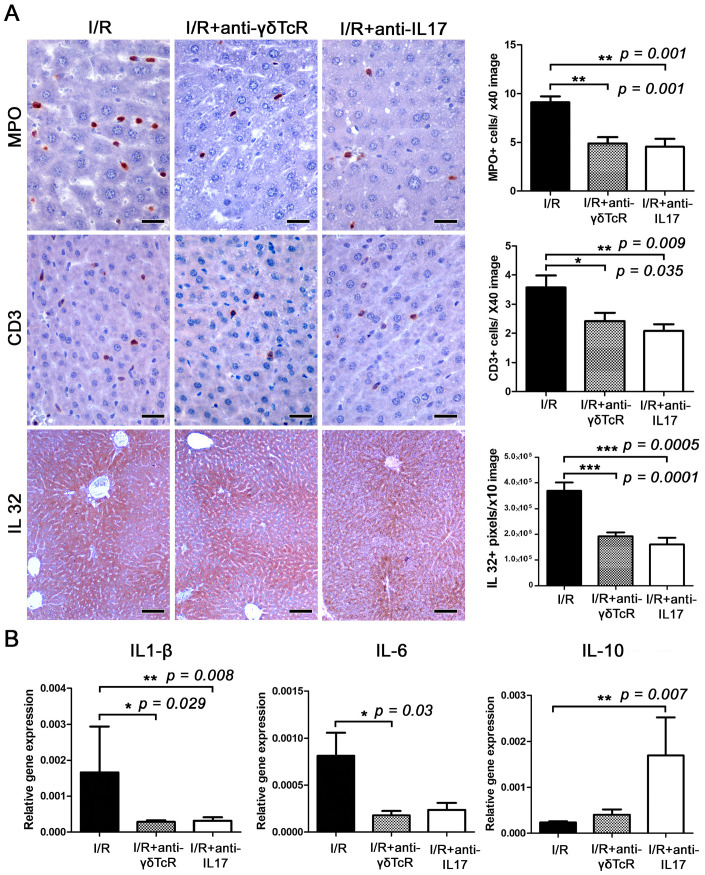
The neutralization of either γδTcR cells or IL17a suppresses I/R-associated inflammation. (**A**) The liver tissue after I/R is infiltrated by MPO+ neutrophils and CD3+ T-lymphocytes. The numbers of MPO+ and CD3+ cells are significantly less in both groups of mice receiving pretreatments. Large amounts of IL-32 localize primarily in the centrilobular zone but often expand to include the whole lobule and create a central-to-central bridging staining pattern. The expression levels of IL-32 are significantly lower in the livers of pretreated mice. (**B**) Quantitative cytokine gene expression analysis of mouse liver shows that pretreatments suppressed the expression of proinflammatory cytokines IL-1β and IL-6 and upregulated the anti-inflammatory cytokine IL-10. The effect was statistically significant. (**A**) Diaminobenzidine chromogen, haematoxylin counterstain. Scale bars: 25 μm (MPO and CD3); 100 μm (IL-32). (**A**,**B**) Numbers on the Y-axis of bar graphs correspond to the mean ± SEM of the parameters assessed. * *p* < 0.05, ** *p* < 0.01*** *p* < 0.001.

Having found that the anti-inflammatory pretreatments we applied reduced IRI in the liver, we sought to examine whether this protection coincided with alterations in local inflammatory networks. For that, we used IHC to label MPO for neutrophils, CD3 for T-lymphocytes and Foxp3 for regulatory T cells in liver sections. Morphometrical counts of IHC-positive cells revealed that both pretreatments worked to reduce MPO-positive (neutrophils) and CD3-positive (T-lymphocytes) cells in I/R-damaged livers at statistically significant levels (Figure 3A). In contrast, Foxp3-positive cells were practically nondetectable in liver sections, suggesting that regulatory T cells did not contribute to the suppression of IRI.

### 3.3. Anti-γδTcR and Anti-IL17 Suppress I/R-Associated Inflammatory Cytokines

This result led us to search for coinciding changes in the cytokine microenvironment in liver IRI. Using IHC and morphometrical counts of IHC-positive signal in liver sections, we found that both the depletion of γδTcR cells and the blockade of IL17a significantly reduced proinflammatory cytokine IL32 (Figure 3A). Subsequently, we investigated gene expression levels of the proinflammatory cytokines TNF-α, IL1-β and IL-6, the anti-inflammatory cytokine IL-10 and the pleiotropic cytokine Tgf-β in the livers of mice. Statistically significant effects of the depletion of γδTcR lymphocytes prior to the induction of I/R included downregulation of IL1-β and IL-6 (Figure 3B). Likewise, the blockade of IL-17a reduced IL1-β and increased IL-10 expression at statistically significant levels (Figure 3B). The differential expression of TNF-α and Tgf-β in the I/R-affected livers of the two experimental groups in comparison to the controls did not reach statistical significance (Figure S1).

### 3.4. Anti-γδTcR and Anti-IL17 Alter the Expression of I/R-Associated Inflammatory and Cell Stress Factors

To further probe the effects of the anti-γδTcR and anti-IL17a pretreatments used on I/R liver injury inflammatory environment, we next assessed the expression of critical molecules with pleiotropic effects, including cellular stress, survival and metabolism, as well as inflammation.

**Figure 4:**
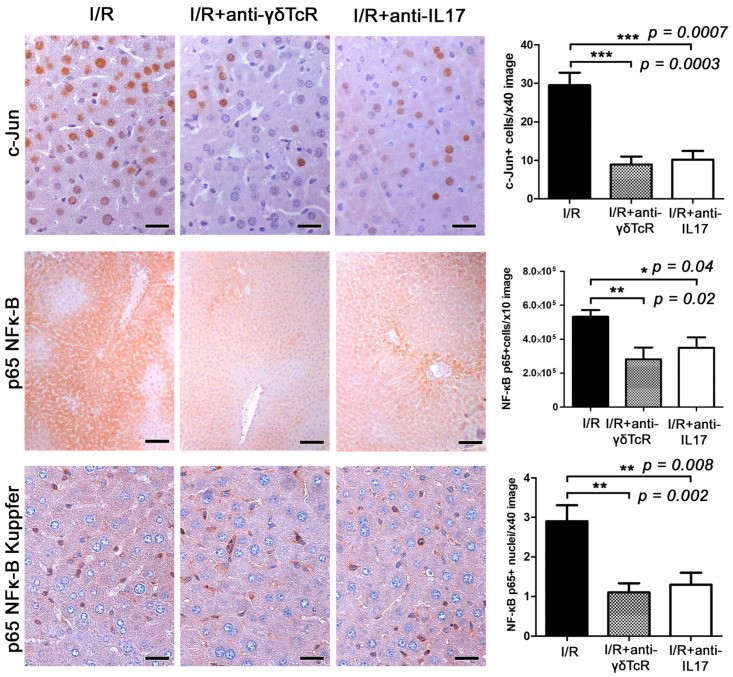
Anti-γδTcR and anti-IL17a pretreatments downregulate c-Jun and NF-κB p65 in the liver of mice with I/R-injury. A high number of cells have a nuclear localization of c-jun in affected liver areas. In comparison with I/R controls, c-jun-positive hepatocyte nuclei are significantly reduced in pretreated mice. Likewise, cytoplasmic NF-κB p65 in hepatocytes is also reduced. In nonparenchymal liver cells comprising mostly Kupffer cells, there is both cytoplasmic expression and nuclear translocation of NF-κB p65. The latter is significantly reduced in the mice receiving pretreatments. Diaminobenzidine chromogen, haematoxylin counterstain. Scale bars: 25 μm (c-Jun and NF-κB p65); 100 μm (NF-κB p65 Kupffer). The Y-axis of bar graphs depicts the mean ± SEM of IHC-labelled cells or pixel counts in high-power magnification images. * *p* < 0.05, ** *p* < 0.01 and *** *p* < 0.001.

For that, we first examined hepatocytes with nuclear localization of the cell stress response nuclear transcription factor c-Jun as an indicator of the extent and severity of I/R-induced hepatocyte stress and damage. c-Jun-specific immunohistochemistry and morphometrical analysis identified statistically significantly fewer hepatocytes with nuclear c-Jun signals in the livers of pretreated mice in comparison to the controls (Figure 4). Likewise, in the pretreated groups, NF-κB p65 presence in hepatocytes due to I/R induction was suppressed at statistically significant levels. NF-κB p65 in hepatocytes was primarily cytoplasmic and its nuclear translocation was unremarkable. An NF-κB p65-positive IHC signal was also evident in nonparenchymal cells of the liver, with the vast majority of them having histomorphology and topographic distribution compatible with Kupffer cells. In these cells, NF-κB p65 nuclear translocation was often identified, especially in areas with prominent I/R-induced lesions. Morphometrical counts of NF-κB p65-positive nuclei in the liver sections of the I/R-treated mice showed that the anti-inflammatory pretreatments worked to reduce NF-κB p65 nuclear translocation in Kupffer cells (Figure 4).

**Figure 5:**
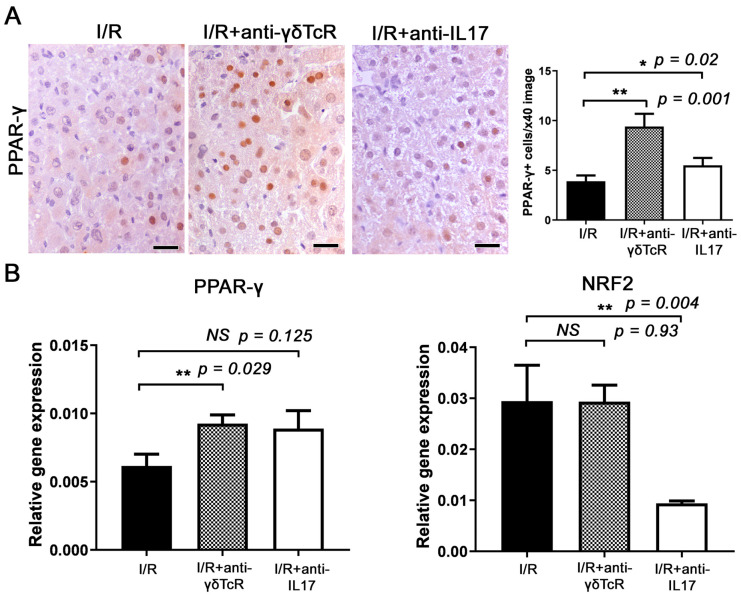
Anti-γδTcR and anti-IL17a increase PPAR-γ and reduce NRF-2 in the livers of mice with I/R-injury. (**A**) Hepatocytes with a nuclear PPAR-γ-positive immunohistochemical signal are more significantly present in the livers of pretreated mice in comparison with the controls. (**B**) Quantitative PPAR-γ gene expression analysis in mouse livers parallels the result of immunohistochemistry. The same analysis shows that the anti-IL17a but not the anti-γδTcR pretreatment correlates significantly with NRF-2 reduction in mouse livers after I/R injury. (**A**) Diaminobenzidine chromogen, haematoxylin counterstain. Scale bars: 25 μm (**A**,**B**) Neutralization of either γδTcR cells or IL17a. * *p* < 0.05, ** *p* < 0.01. NS, Nonsignificant.

We also assessed PPAR-γ, a nuclear receptor with important roles in immune response and inflammation. By immunohistochemistry and morphometric counts, we found significantly more hepatocytes with PPAR-γ expression (Figure 5A) in the livers of mice that received the anti-inflammatory treatments prior to I/R. Quantitative gene expression analysis for PPAR-γ supported this observation. However, in comparison with the controls, the upregulation of PPAR-γ reached statistical significance only for the anti-γδTcR-pretreated and not for the anti-IL17a-pretreated group (Figure 5B).

Finally, we investigated the key transcription factor NRF2, which activates antioxidative stress responses following inflammation and tissue damage. Quantitative gene expression analysis showed that the IL17a neutralization downregulated NRF2, whereas the depletion in γδTcR cells did not affect its expression (Figure 4B).

## 4. Discussion

Both IL17a and γδ-Τ cells seem to be important mediators in hepatic IRI. There is a growing literature highlighting their role in intestinal [28], renal [29,30] and CNS [31] IRI. Savransky et al. showed that a deficiency in γδ-T cells led to reduced mortality and structural injury during renal IRI [30]. In brain and liver I/R models, they have been identified as a source of IL17a and RORγt expression [32,33,34]. We also observed the attenuation of liver IRI after IL17a and γδTcR blockade, as evidenced by a decrease in markers for histopathological injury and decreased lipid peroxidation compared to the controls.

After IRI, neutrophils and T cells are recruited in the liver as a result of cytokine and adhesion molecule production by Kupffer cells [35,36,37,38]. The role of neutrophils in liver injury via ROS production is known [39]. Caldwell et al. showed that the extent of neutrophil recruitment to the liver was reduced by anti-IL17a in a dose-dependent fashion [40]. Other groups have also supported the role of IL17a in neutrophil recruitment [11,41,42]. Additionally, γδTcR has been implicated in neutrophil recruitment in psoriatic arthritis [43] and burns [44]. Fabrega et al. observed a rise in IL17/IL23 in the blood after liver transplantation, implying that Th17 cells play an important role during liver transplantation and rejection [45]. The role of regulatory T cells (Tregs) has not been clarified as yet. Lu et al. suggested that Tregs mitigate the inflammatory response and minimize tissue injury in liver I/R [46], while Kuboki et al. found no effect [47]. In our experiments, anti-γδTcR and anti-IL17a pretreatments resulted in decreased MPO and CD3-positive cells, indicating a negative impact on the recruitment of neutrophils and effector T cells, respectively. Foxp3-positive cells were practically nondetectable in liver sections after IRI, suggesting that Treg cells did not contribute to the suppression of the I/R pathology. Taken together, these results indicate a significant role for both γδTcR and IL17a in liver IRI through the migration of neutrophils and T cells.

Studies by other groups have shown the importance of IL-32 in upregulating other proinflammatory cytokines, including IL1β, IL6 and TNFα, and increasing hepatic IRI [27,48]. The protective effect of the inhibition of either γδTcR or IL17a observed in this study also coincides with the decreased expression of IL-1β, IL-6, and IL-32. This reduction may also contribute to the depletion of effector T cells and Treg cells, as IL1β and IL6 have been implicated in their differentiation [49,50]. Treg cells have been considered to be a major source of endogenous IL10 [51], which has been shown to play a protective role in liver IRI [52]. We observed that IL10 levels were significantly upregulated following IL17a, but not as an anti-γδTcR blockade. This suggests that IL17a may suppress IL10 expression.

Likewise, anti-γδTcR or anti-IL17a pretreatment in our study downregulated the transcription factor c-Jun in hepatocytes, and NF-κB in both hepatocytes and Kupffer cells, compared to the controls. Yang et al. showed that NF-κB activation was decreased in IL17a knockout mice after liver I/R injury, indicating that IL17a was required for the activation of NF-κB, further leading to reduced apoptosis and inflammation [53]. Our study is in line with these findings, indicating that the NF-κB signalling cascade is an important downstream effect of IL17a and γδ-Τ-cell activation. Yoshidome et al. also suggested that IL10 plays a protective role in inflammation by downregulating NF-κB [54]. In contrast, our results suggest that a γδTcR blockade downregulates NF-κB independently from IL10, implying an alternative regulatory pathway.

Furthermore, we examined the expression of two transcription factors with a protective role in I/R injury, PPAR-γ and NRF2. PPAR-γ is highly expressed in macrophages and its activation has been linked to promoting the anti-inflammatory macrophage phenotype, leading to a decreased inflammatory response [55,56]. Linares et al. showed that the PPAR-γ pathway could be a protective autoregulatory mechanism to counter the inflammatory response following I/R injury [57]. Our study also shows that anti-γδTcR and anti-IL17a pretreatment resulted in the increased expression of PPAR-γ, suggesting that this pathway could also play a role in the γδTcR and IL17a protective effect. NRF2 expression has been implicated in protecting cells from oxidative stress in response to surrounding increased ROS production and endoplasmic reticulum stressors, specifically in liver I/R injury [16]. Its expression seems to be independent of γδTcR signalling; however, the IL17a blockade resulted in the downregulation of this protective transcription factor.

The role of γδ-T cells in hepatic IRI has long been neglected. These results build on existing knowledge and establish a role for γδ-Τ cells as important mediators of cell injury in hepatic IRI, particularly with regard to neutrophil and T-cell recruitment and NF-κB downregulation, further placing the activation of γδTcR among the initial steps of hepatic I/R pathogenesis. Our results indicate that there is significant crosstalk between γδTcR and IL17a, as both contribute to IRI through similar pathways, i.e., altering cytokine production and similarly influencing the expression of transcription factors.

Our study has some limitations, namely the complex crosstalk between γδTcR and IL17a, the use of CD3, which is a universal T-cell marker, not differentiating T-cell subgroups, and the fact that we have not explored the role of RORγτ in conjunction with these two pretreatments. These limitations can form the basis of further studies.

## Figures and Tables

**Table 1:** Primers used for gene expression analysis.

Primer	Sequence (5′-3′)	GenBank Access No	Positions	Amplicon Size (bp)	Primer Conc. (nM)	Annealing T (°C)
IL1-β-F	TGGACCTTCCAGGATGAGGACA	NM_008361.4	331–352	148	100	61
IL1-β-R	GTTCATCTCGGAGCCTGTAGTG		457–487			
IL6-F	TACCACTTCACAAGTCGGAGGC	NM_031168.2	189–209	144	250	60
IL6-R	CTGCAAGTGCATCATCGTTGTTC		315–332			
IL10-F	TCAGCCAGGTGAAGACTTTCT	NM_010548.2	210–230	147	200	63
ILA10-R	GGGGCATCACTTCTACCAGG		337–356			
PPAR-γ-F	GTACTGTCGGTTTCAGAAGTGCC	NM_011146	618–640	104	250	60
PPAR-γ-R	ATCTCCGCCAACAGCTTCTCCT		698–719			
NRF2-F	CAGCATAGAGCAGGACATGGAG	NM_010902	765–786	107	250	60
NRF2-R	GAACAGCGGTAGTATCAGCCAG		850–871			
TNF-α-F	GTCCCCAAAGGGATGAGAAG	NM_013693.3	337–356	134	300	59
TNF-α-R	CACTTGGTGGTTTGCTACGA		451–470			
TGF-β-F	GGAGAGCCCTGGATACCAAC	NM_011577.2	1697–1716	149	250	59
TGF-β-R	GCAGGGTCCCAGACAGAAG		1827–1845			
GAPDH-F	CATCAAATGGGGTGAGGCCG	NM_001289726.1	338–357	149	250	63
GAPDH-R	CCATCACAAACATGGGGGCA		467–486			

## Data Availability

All data in this study are available from the corresponding author upon request.

## Supplementary Materials

The following supporting information can be downloaded at: https://www.mdpi.com/article/10.3390/jcm12051751/s1, Figure S1: The depletion of either γδTcR cells or IL17a prior to I/R induction did not induce significant differences in the expression of both TNF-α and Tgf-β in the liver of mice. The Y-axis of the bar graphs depicts the mean ± SEM of relative gene expression. NS, Nonsignificant.
