# Supplementary material for: Inhibition of γδ-TcR or IL17a Reduces T-Cell and Neutrophil Infiltration after Ischemia/Reperfusion Injury in Mouse Liver
Source: J Clin Med. 2023 Feb 22;12(5):1751. doi: 10.3390/jcm12051751 (PMC10002490; doi:10.3390/jcm12051751)
Supplement: Supplementary file 1 [file jcm-12-01751-s001.zip › jcm-2192769-supplementary.pdf]

Supplementary Materials

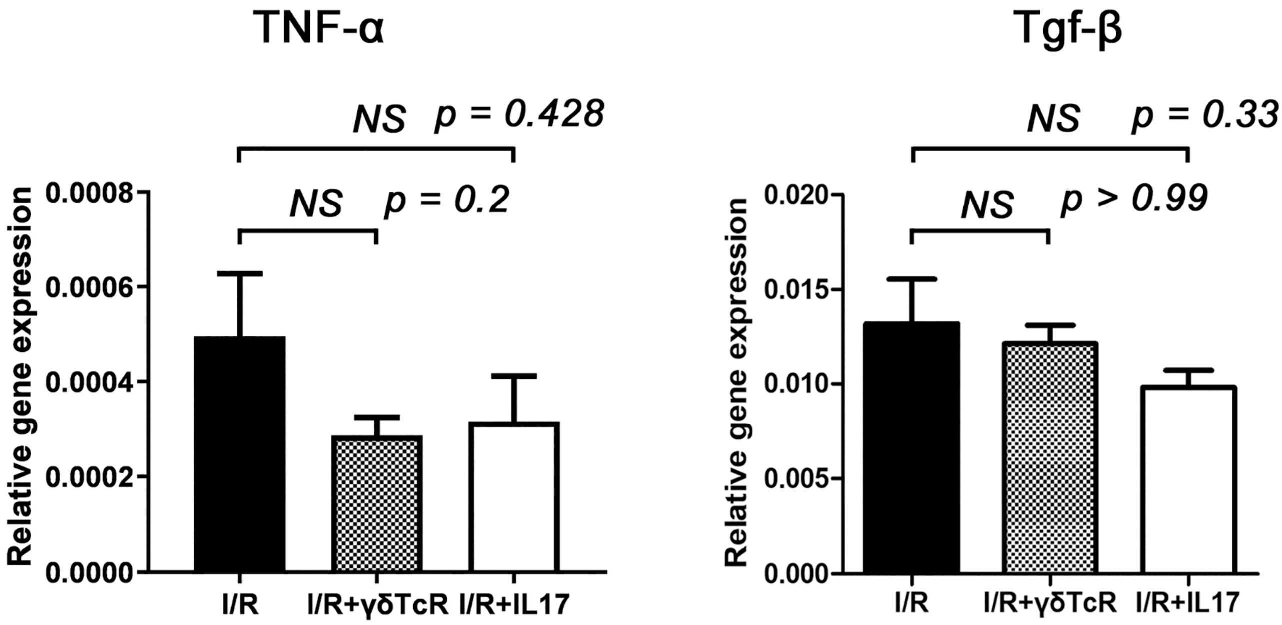

Figure S1. The depletion of either γδTcR cells or IL17a prior to I/R induction did not induce significant differences in the expression of both TNF-α and Tgf-β in the liver of mice. The Y-axis of the bar graphs depicts the mean ± SEM of relative gene expression. NS, Nonsignificant.
